# A Comprehensive Review on Social Inequalities and Pregnancy Outcome—Identification of Relevant Pathways and Mechanisms

**DOI:** 10.3390/ijerph192416592

**Published:** 2022-12-10

**Authors:** Valentin Simoncic, Séverine Deguen, Christophe Enaux, Stéphanie Vandentorren, Wahida Kihal-Talantikite

**Affiliations:** 1 LIVE UMR 7362 CNRS (Laboratoire Image Ville Environnement), University of Strasbourg, 67100 Strasbourg, France; 2 Equipe PHARes Population Health Translational Research, Inserm CIC 1401, Bordeaux Population Health Research Center, University of Bordeaux, 33076 Boedeaux, France; 3 Santé Publique France, French National Public Health Agency, 94410 Saint-Maurice, France

**Keywords:** social determinants, neighborhood, birth outcomes, social inequalities

## Abstract

Scientific literature tends to support the idea that the pregnancy and health status of fetuses and newborns can be affected by maternal, parental, and contextual characteristics. In addition, a growing body of evidence reports that social determinants, measured at individual and/or aggregated level(s), play a crucial role in fetal and newborn health. Numerous studies have found social factors (including maternal age and education, marital status, pregnancy intention, and socioeconomic status) to be linked to poor birth outcomes. Several have also suggested that beyond individual and contextual social characteristics, living environment and conditions (or “neighborhood”) emerge as important determinants in health inequalities, particularly for pregnant women. Using a comprehensive review, we present a conceptual framework based on the work of both the Commission on Social Determinants of Health and the World Health Organization (WHO), aimed at describing the various pathways through which social characteristics can affect both pregnancy and fetal health, with a focus on the structural social determinants (such as socioeconomic and political context) that influence social position, as well as on intermediary determinants. We also suggest that social position may influence more specific intermediary health determinants; individuals may, on the basis of their social position, experience differences in environmental exposure and vulnerability to health-compromising living conditions. Our model highlights the fact that adverse birth outcomes, which inevitably lead to health inequity, may, in turn, affect the individual social position. In order to address both the inequalities that begin in utero and the disparities observed at birth, it is important for interventions to target various unhealthy behaviors and psychosocial conditions in early pregnancy. Health policy must, then, support: *(i)* midwifery availability and accessibility and *(ii)* enhanced multidisciplinary support for deprived pregnant women.

## 1. Introduction

A growing body of evidence confirms that lived experiences during the first thousand days of life can be a critical determinant of a child’s likelihood of survival, growth, and well-being during his/her entire life. With a range of short- and long-term consequences, adverse birth outcomes include low birthweight (LBW) and preterm birth (PTB). LBW is defined by the WHO as a birthweight of below 2500 g (referenced P07.0–P07.1 in the 10th revision of the international classification of diseases–ICD 10). PTB is defined as childbirth occurring at less than 37 completed weeks or 259 days of gestation (referenced P07.2–P07.3 in ICD 10) [1]. These outcomes continue to represent a major public health issue, and the consequences of LBW and PTB include fetal and neonatal mortalities as well as morbidities, including poor cognitive development and an increased risk of chronic disease later in life [1,2,3]. Recent studies have demonstrated that LBW increases the risk of diabetes and cardiovascular disease during adulthood [4]. Moreover, it is well documented that, in comparison with children born at term, those born prematurely are more likely to present cerebral palsy, sensory deficits, learning disabilities, and respiratory illnesses [5,6,7,8,9,10,11,12,13]. Complications related to PTB are the leading causes of death for children aged under 5, resulting in about a million deaths worldwide in 2015 [14,15]. Moreover, adverse consequences related to LBW and PTB contribute very significantly to global health costs [15,16,17,18,19]. According to the European Union (EU) benchmarking report of 2009/2010, statistical data collected from 14 European countries demonstrate the significant and growing cost of prematurity in Europe [20].

The WHO estimates that between 15% and 20% of births worldwide are LBW, representing 20 million births a year [16]. It is also estimated that more than 15 million babies are born preterm every year—more than 10% of babies worldwide [5]. In developed countries, PTB rates have been reported as ranging from 5% to 7% of live births [21], and these figures appear to be on the rise [22]. According to the European Perinatal Health Report, LBW babies accounted for less than 4.5% of all births in Iceland, Sweden, and Finland and around 10% in Spain and France. In some countries, the percentage of LBW babies was significantly higher in 2015 than it was in 2010. PTB rate comparisons for 2010 and 2015 differed widely between countries and were significantly higher in eight countries [23].

The wide literature has indicated that the healthy development of the child during the first thousand days is strongly related to maternal health status during pregnancy, living and working conditions, and neighborhood characteristics. More specifically, maternal health status during pregnancy (including excessive gestational weight gain (EGWG), gestational diabetes mellitus (GDM), and obesity) is known to have significant consequences for newborn mortality and morbidity [18], including preterm birth [19]. The healthy development of a child during the first thousand days, therefore, depends on both a healthy mother during pregnancy and a healthy pregnancy. Although their etiology is thought to be multifactorial [5], PTB and LBW risk factors are still not understood completely. During the first thousand days of life, then, pregnant women, fetuses, and newborns are exposed daily and simultaneously to a multitude of factors, including maternal or fetal medical conditions, genetic influences, infertility treatments, behavior, iatrogenic prematurity, community resources, and environmental exposure [22,24,25]. The literature supports the idea that maternal and prenatal nutrition can—by providing the essential building blocks for brain development, healthy growth, and a resistant immune system—affect a child’s ability to grow, learn and thrive.

Beyond the factors described above, this literature further suggests that maternal, parental, and contextual characteristics may also affect the pregnancy and health status of fetuses and newborns. 

Over the past decade, a growing body of evidence has shown that, at both individual and aggregate levels, social determinants are an important determinant of child health, including during the first thousand days of life. Numerous studies have found that social factors, including maternal age and education, marital status, pregnancy intention, and socioeconomic status, have been linked to poor birth outcomes. In addition, some studies suggest that beyond social factors at individual and contextual levels, the living environment or “neighborhood” has emerged as an important determinant through which to investigate health inequalities, particularly for pregnant women.

The aim of our paper, then, was to comprehensively review published studies addressing social inequalities in adverse birth outcomes. Our work is mainly divided into three steps:

(a) to outline birth outcomes by social determinants and neighborhood deprivation and describe both methodological approaches and potential confounders.
(b) to summarize the effect of socioeconomic characteristics (at individual and neighborhood levels) on pregnancy outcomes in selected studies.
(c) to propose a theoretical model on the pathways and possible mechanisms through which social determinants may be related to adverse pregnancy outcomes.

## 2. Material and Methods

### 2.1. Search Strategy

We searched the PubMed public health database for English-language articles published between January 2000 and April 2022 using relevant title expressions and reviewed the reference lists of the selected articles. We also screened papers citing the selected articles. 

The search strategy followed the Preferred Reporting Items for Systematic Reviews and Meta-Analyses (PRISMA) guidelines and was performed with the following keywords found in article titles and/or abstracts: “deprivation,” “socio-economic,” “socioeconomic,” “inequality,” “inequalities,” “disadvantage (s),” “disadvantaged,” “advantage (s),” “advantaged,” “income,” “Neighbor-hood,” “employment,” “neighborhood,” “lifestyle,” “socio-occupational,” “insurance,” “educational,” “social,” “healthcare.” These terms were used in conjunction with the following other title terms: “birth outcomes,” pregnancy outcome,”” low birth weight,” “birth weight,” “low-birth-weight,” “birthweight,” “birth-weight,” “preterm birth,” “gestational age,” “LBW,” and “PTB.”

### 2.2. Studies Selection Strategy

In the first step, the inclusion criteria were human studies, peer-reviewed papers written in English, and articles published after 2000. We restricted our review to pregnant women and pregnancy outcomes. Papers presenting non-original studies were ultimately excluded.

In the second step, the inclusion criteria were specific pregnancy outcomes definitions, including birthweight, low birthweight, preterm birth, or small for gestational age (SGA). Secondary criteria were studies investigating specific social determinants.

Two authors (VS and WK) independently screened the papers based on information in the title, the first pool of articles consisted of 359 results, then abstracts and full manuscripts were screened to select those papers considered relevant based on the screening criteria described below. 

Data extraction for each study, we extracted and reported in several tables the following information:

- General information: first author’s name, country of origin, and date of the study.
- Main study characteristics: study design, period, location, statistical methods, population size, and main findings (related to PTB, LBW, BW, and SGA outcome).
- Participant characteristics: information on confounders.
- Outcome measures.

Assessments of the association, including odds ratios (ORs), hazard ratios (HRs), relative risks (RRs), and other metrics measuring the strength of association between outcomes and social determinants, were extracted. When several measures of association were available, we reported those from the fully adjusted models.

The two authors (VS and WK) independently extracted all data from selected studies.

The present study has provided some additional empirical support for the potential role played in adverse birth outcomes by social determinants. Using the conceptual framework for action on the social determinants of health provided by the Commission on Social Determinants of Health and the WHO, we began by adapting a conceptual model of the mediating variables associated with socioeconomic deprivation and their hypothetical relationship with pregnancy outcomesSecondly, we proposed a theoretical framework describing hypotheses on the underlying mechanisms that might explain the association between social deprivation and maternal, fetal, and newborn health.

## 3. Results

From the 359 studies screened, 35 carried out since 2000 met our selection criteria and investigated the social inequalities of adverse pregnancy outcomes, including LBW, PTB, and SGA (defined as birthweight or length below the 10th percentile according to standard percentile charts for sex and gestational age in the population, referenced in the ICD10: O36.5, P05.0, P05.1). 

### 3.1. Location and Population

Most of these studies were conducted in the United States (both north and south) [26,27,28,29,30,31,32,33,34,35,36,37,38,39,40,41,42,43,44,45,46,47,48,49]. We also included six studies conducted in Europe and the United Kingdom [50,51,52,53,54,55]. Just one was conducted in Asia [56], and another study covers four countries (US, Canada, UK, and Australia) [57]. Our selection includes a diverse range of study designs. While a majority are cohort studies [29,31,35,37,38,42,44,45,50,54,57,58], others are birth record or population-based [30,32,36,39,39,40,51], cross-sectional [26,28,33,34,41,43,46,47,48,52,53,56,59], ecological [49] and case-control [55] studies.

### 3.2. Adverse Pregnancy Outcomes 

Several studies investigated birthweight [35,43,51,52] or gestational age but most investigated specific pathological outcomes. Several investigated LBW and subtypes [29,32,37,38,39,41,45,50,51,53,56,57], while others investigated PTB and subtypes [28,31,33,34,36,37,38,39,40,42,44,46,47,50,51,54]. Lastly, some studies investigated SGA [26,28,30,38,46,50,52,58].

### 3.3. Methodological Approaches

In this section, we formulate general considerations on methodological issues that appear relevant to investigating the effects of sociodeterminants on birth outcomes.

### 3.4. Assessment of Social Inequalities 

Differences between the statistical methods implemented may obscure the comparison of the findings. Conventional approaches (such as logistic regression) allocate contextual effects to the individual level, whereas multilevel analysis strives to distinguish individual socioeconomic health effects from those estimated at the places where they live. This approach is now acknowledged as the gold standard for assessing the true health-related contextual effect [46]. However, most of the studies applied more classical multivariate regressions, such as logistic, binomial, and multinomial regression [32,37,38,40,41,42,43,44,46,47,48,50,51,52,54,55,56,57,58,59], while just five studies performed multilevel analysis [26,30,33,34,35]. Two studies tested the mediation hypothesis using a structural equation model [44,45]. In the first of these, the authors investigated the multiple pathways through which social determinants can impact birth outcomes [44], while the second sought to estimate the extent to which perceived stress could mediate the effects of neighborhood amenities on depressive symptoms during pregnancy [45]. 

### 3.5. Various Confounders 

Because of weaknesses that could affect the strength of the measure of association and, consequently, the formulation of accurate conclusions, caution is needed in interpreting and comparing study findings. In particular, the various confounding factors may render comparisons between studies difficult. As is often the case in environmental epidemiology, most studies consider maternal characteristics such as age, ethnicity, marital status, level of education, or parity to be socio-demographic confounders (with the exception of five studies [43,48,49,55,59]). Specifically, a large proportion of these studies examined maternal anthropometric characteristics [28,29,35,39,40,42,44,45,46,50,54,58] such as height, weight, and body mass index (BMI), and healthy or unhealthy behaviors [28,29,30,31,34,35,39,40,43,44,45,48,50,55,56,57,58,59] such as physical activity, smoking or alcohol consumption. Some studies explored chronic disease [30,31,34,42,56,58] or pregnancy complications [28,31,34,37,42,44,46,56,58]. Others adjusted on newborn characteristics, such as gestational age at birth, birth date, or sex [29,35,38,42,43,50,51,52,56,57]. 

It is possible that newborn and maternal levels of adjustment may not be enough to capture the full complexity of multifactorial exposure, and this could lead to heterogeneous or insignificant results. Some studies therefore adjusted neighborhood characteristics such as place of residence or unemployment level in the area [28,33,34,41,46,53]. However, even when these studies did investigate the influence of neighborhood characteristics, none adjusted on household characteristics such as family income, parental life course factors, and family smoking status (other studies have explored these factors independently: [31,38,45,48,59]). In the same way, none of these studies have explored paternal characteristics (only one study, unlike all previous studies, investigated these factors [52]). The complexity of the links between these various factors and the potential feedback effect of one variable on another suggests that it is important to study all these adjustment variables in order to limit exposure bias and homogenize the results.

### 3.6. Definition of Social Determinants

Levels and characteristics of socioeconomic inequality differ between studies, depending on the data used. Some authors relied on individual socioeconomic or socio-demographic characteristics alone [26,29,31,37,38,44,47,50,52,54,55,58], such as maternal education level [26,31,32,33,34,35,37,38,39,42,43,44,45,47,48,50,52,54,55,56,57,58], paternal education level [34,38,52], employment [37,55,58], income [26,38,46], etc. Others used neighborhood socioeconomic characteristics only [28,30,51,53], such as neighborhood income index [28,30,32,35,40,46,48,51,55,57], unemployment [33,35,53] and education level [35,48,51]. Most studies, however, used both individual and neighborhood socioeconomic characteristics [32,33,34,35,39,40,42,43,45,46,48,51,57]. Some authors used family or housing socioeconomic factors [41,44,45,46,48,55,56], such as family income [31,38,48,55,56], grandmother’s educational level [45], or grandmother in the household [41]. The main characteristics of these studies are summarized in Table 1.

### 3.7. Findings in Terms of Social Determinants and Health Inequalities

Individual socioeconomic level and birthweight—Given the heterogeneity of the variables used to describe socioeconomic conditions (including socio-demographic, socioeconomic, and other variables), we will hereafter use the term socioeconomic conditions to refer to social determinants. Most studies showed an increased risk of LBW (or decreased birthweight) for pregnant women having adverse socioeconomic conditions.

For instance, in terms of the maternal level of education, LBW risk varied between OR = 2.0 [1.49–2.71] [55] and RR = 3.77; 95%CI [2.26–6.30] [38]. The strength of association with unemployment during pregnancy is: OR = 1.7; 95%CI [1.37–2.10] [55], maternal income: OR = 1.7; 95%CI [1.58–2.84] [55] or level of employment precarity: RR = 1.48, 95%CI [1.11–1.98] [29]. In addition, when comparing mothers with lower vs. higher levels of education, the decrease in birthweight was equal to beta = −103; 95%CI [−108; −97] [52]. In terms of paternal education level, the decrease was lower, though still significant, equal to beta = −56; 95%CI [−61; −50] [52]. 

Only one study investigated the impact of the interaction between several unfavorable maternal socioeconomic conditions and the effect on LBW. It revealed that the chances of delivering an LBW baby increased when the interaction between education level, marital status, and income were taken into account. More specifically, the OR reached 7.8; 95%CI [4.32–14.06] for pregnant women accumulating a low level of education, low income, and unstable marital status in comparison with other women. Moreover, being unemployed during pregnancy combined with a low level of education was at 4.7; 95%CI [3.35–6.47] a higher risk of delivering an LBW baby in comparison with others. The same study also found that low income interacting with unemployment during pregnancy significantly increased LBW risk (OR = 3.6; 95%CI [2.85–4.65]) [55].

In addition, (though less frequently), other studies looked at whether the maternal early-life socioeconomic environment could be linked to LBW. Huang et al. found an association between a grandmother’s education at the time of a mother’s birth and the birthweight of her grandchild, independent of the mother’s mediating life course (beta = −54 g 95%CI [−14.0; 122.1]) per increase in educational level [45]. 

Socioeconomic deprived neighborhood and birthweight—Most studies investigating the effect of socioeconomic conditions (measured at the neighborhood level on birthweight) confirmed the existence of social and territorial health inequalities [32,39,43,51,53,57]. However, a few studies [35,48,49] suggested that from fully adjusted models (including both individual- and family-level characteristics), the effect of neighborhood socioeconomic status was not as important as previously demonstrated [35,48]. For instance, a significant decrease in birthweight was observed in the most deprived areas, in comparison with the least deprived (Beta = –113.4 g (95%CI [ –133.0; –93.8]) [51]. Martinson et al. found that the risk of LBW increased as family income fell (for bottom income quartile: OR = 2.37; 95%CI [1.80;3.11], OR = 1.78; CI95% [1.30;2.44] and OR = 2.11 95%CI [1.12;3.99]) in the US, UK, and Australia, respectively [57]. The risk of LBW increased far more among African American women living in low-poverty neighborhoods than it did for white women living in low-poverty neighborhoods (OR: 5.23; 95%CI [2.26;12.10]) in the study by Wallace et al. [39].

Individual social position and preterm birth—As described above for birthweight, most studies confirmed that the risk of PTB was associated with individual or family socioeconomic characteristics [31,33,36,37,38,39,42,44,46,47,50,54]; all but four studies reached this conclusion [37,39,50,54]. For instance, the risk of PTB is significantly linked with a low level of maternal education (compared to those having a higher level of education), with a risk of about 1.2 in Kaufman et al. [33] and El-Sayed et al. [47]. To a lesser extent, in Dunlop et al., the strength of association is about 1.1 [42], while the risk of PTB exceeded two in Filho et al. [38]. Family income/occupation is also recognized as increasing the risk of PTB; significantly, the risk of PTB is twice as high among families on lower incomes or among family heads having less skilled occupations [38].

Socioeconomically deprived neighborhood and PTB—In terms of birthweight, most studies (all but two [33,42]) revealed a socioeconomic relationship to PTB outcome [28,34,36,39,40,46,51]. More specifically, the risk of PTB is increased among women residing in counties within the highest quartile of poverty, compared with the lowest quartile (OR = 1.18;95% CI [1.03,1.35] [34]), the risk of PTB is also higher among women residing in deprived neighborhoods, compared with the most privileged neighborhoods (OR = 1.16;95%CI [1.06–1.27] [46]. Furthermore, the risk is higher among the most deprived groups, compared with the most privileged groups (OR = 1.5;95%CI [1.3;1.7]), for 1991–2000) [51]. Some studies suggested that minority mothers living in poor areas were at greater risk of PTB [39,40].

Individual social position and small for gestational age (SGA)—All but two of the studies [30,50] suggested that SGA was associated with social characteristics [26,28,30,38,46,50,52,58] measured at the family or parental level. The risk of SGA is close to 1.3 in families or parents who are unemployed, compared with those who are not [58], while the strength of the association reaches 2.8 in families where the father or mother has a low education level [38].

Family or parental income and occupation were also identified as significant risk factors for SGA. In the study by Shankardass et al. OR was equal to 2.0; 95%CI [1.78;2.26] for the lowest income decile [46]. In the study by Filho et al., the risk of SGA increased where the head of the family had a less skilled occupation (RR = 1.79 95%CI [1.29;2.47]) [38] in comparison with others.

Socioeconomically deprived neighborhood and small gestation age (SGA)—Few studies have investigated the question of the socioeconomically deprived neighborhood as a risk factor for SGA [28,30,46]. Results have tended to confirm the existence of social health inequalities such as those described for birthweight and PTB; it is only in the case of Shankardass et al. that a significant increase in SGA risk was noted in the most deprived neighborhoods (OR = 1.18; 95%CI [1.07;1.30]) [46].

### 3.8. Framework for Action on Social Determinants of Health

**Figure 1:**
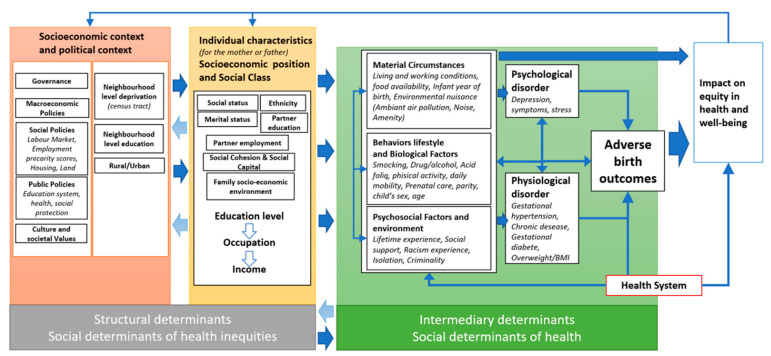
A conceptual framework adapted and modified from the WHO’s Commission of Social Determinants of Health and from the analysis of the studies reviewed. Amenity is a useful or enjoyable feature. An environmental amenity is referred to any benefits that increase the attractiveness of a place or neighborhood by increasing its comfort or convenience. Examples of amenities are pleasant views, good schools or good green space.

Figure 1 provides a holistic view of inequities in newborn health. The conceptual framework has two main components: (i) Structural determinants (including socioeconomic, socio-demographic, and political context as well as socioeconomic position and social class) and (ii) Intermediary determinants.

(i). Structural determinants

These factors create or enhance social stratification and contribute to defining individual social positions. These mechanisms determine the health opportunities available to different social groups, as well as their access to various resources.

Firstly, context can play a role in health inequalities. As the WHO has stated, the inclusive term “context” covers all the social and political mechanisms that generate, configure, and maintain social hierarchies. This includes the labor market, educational system, and political institutions, as well as other cultural and societal values. We have also learned that neighborhood contextual aspects can affect inequities in health; in this case, we speak of neighborhood-level deprivation or neighborhood-level of education. Several studies suggest an association between pregnancy outcomes and such structural determinants as neighborhood-level deprivation [34,39,40,46,51,53] or neighborhood unemployment rate [53].

Secondly, social position matters. In general, very few studies in the literature consider all characteristics –studies including equity assessment frequently use income, education, and occupation as a proxy for social position. Social conditions are also closely associated with age, ethnicity, or housing characteristics (marital status, partner employment, partner education). [60]. Since each of these indicators covers a different aspect of social stratification in terms of access to information, material goods or privileges, and social skills, it is preferable to use as many as possible rather than just one. [61]. Many studies have suggested an association between pregnancy outcomes and individual socioeconomic characteristics, such as maternal or paternal education level [33,38,42,47,52,55,57,58], maternal employment [29,58], socioeconomic status [26], low maternal income [55] and ethnicity [39,44].

Socioeconomic position can be meaningfully measured at various scales and at both individual and household levels. Each level may independently contribute to the distribution of exposure and pregnancy outcomes. Some studies tend to show significant findings at the household level, such as family income [38,46] or heads of the family having less skilled occupations [38].

Social position can also be measured at different points in the lifespan (e.g., infancy, childhood, adolescence, and adulthood, in the current moment or past 5 years, before or during pregnancy, etc.). Relevant time periods depend on presumed exposures, causal pathways, and associated etiologic periods [62]. Some studies reviewed here have suggested that maternal early-life socioeconomic environment is directly associated with PTB [44], while others have shown the paternal census track of residence during childhood to be associated with family income [26].

Together, context (neighborhood and household level), structural mechanisms, and individual social position constitute the “social determinants” of health inequities; action on these determinants is aimed at achieving an equitable distribution of health, well-being, and adverse pregnancy outcomes across social groups. It is also important to keep in mind that phenomena related to social position can also influence aspects of context, as suggested by the arrows pointing back to the left.

Social position influences health through more specific intermediary determinants. Individuals experience differences in environmental exposure and vulnerability to health-compromising conditions as a result of their respective social status, which, in conjunction with intermediary factors, directly affects the level and frequency of environmental exposure as well as the level of vulnerability. Differences in exposure can also generate more or less vulnerability within the population after exposure [60].

(ii). Intermediary determinants

Intermediate factors are partly a consequence of social configuration, and when they accumulate, this determines differences in both environmental exposure and vulnerability to health-compromising conditions. The social determinants of health inequities are linked to a set of individual-level influences, including environmental exposure, living conditions, biological and physiological factors, and health-related behaviors.

The health system should be viewed as an intermediary determinant in its own right and is closely linked to organizational models of health service delivery. The health system can directly address differences in exposure and vulnerability both by improving equitable access to care and by promoting intersectoral action aimed at improving health status. [60]

The health sector uses five types of actions to tackle health inequalities:

- Public health prevention actions, may reduce inequalities by means of actions in various domains, such as nutrition, sanitation, housing, and working conditions
- Actions such as vaccination, empowerment, and social support as factors in building resistance to the health effects of unevenly distributed exposures
- Treatment and rehabilitation actions for those health problems that constitute the socioeconomic gap in the disease burden (rehabilitation of disabled people)
- Policy actions aimed at reproducing the contextual factors (e.g., social capital) capable of mitigating the effects of poverty on health
- Protective actions against the social and economic consequences of ill health by means of health insurance, sickness benefits, and labor market policies [63].

The unequal distribution of these intermediary factors (whether alone or combined with differences in exposure, vulnerability to health-compromising conditions, or differential consequences of ill health) constitutes the primary mechanism through which social position generates health inequities. The model includes the health system as a social determinant of health and illustrates the health sector’s ability to influence the process in three ways, namely by acting upon differences in environmental exposures, differences in vulnerability, and differences in the consequences of illness for people’s health, as well as for their social and economic circumstances.

One other important element is that the health sector plays a key role in the promotion and coordination of policy as regards interventions aimed at altering both differential exposures and differential vulnerability by taking action on intermediary factors such as material circumstances, psychosocial factors, and behavioral/biological factors [60].

Social Cohesion & Social Capital: Social capital can be defined as social organization features (such as networks, norms, or social trust) that facilitate coordination and cooperation for mutual benefit [64]. Researchers have claimed that social capital is a key factor in shaping population health [64,65,66]. For the population of pregnant women, one element of social cohesion could, for instance, be the level of the grandmother’s education and the presence of a grandparent in the household during pregnancy and pre-pregnancy. One study stresses the important role played by grandmothers in Black families, where their presence is associated with healthier pregnancies. In addition, for Black pregnant women who were poor in childhood, living with a grandmother reduces the risk of low birthweight [41,45].

#### Gradients and Feedback Effect

It is important to bear in mind that the association of social position and health occurs along a gradient at every level of the social scale—not just below the poverty threshold [67]. The model also highlights the reverse (or feedback) effects through which adverse health outcomes (and adverse birth outcomes in particular) inevitably lead to inequity in health and altered well-being; this may, in turn, affect an individual’s social position. It should also be noted that widely prevalent diseases can affect key social, economic, and political institutions.

### 3.9. A Theoretical Contribution Aimed at Gaining a Better Understanding of the Potential Social Deprivation Effect on Maternal and Newborn Health

Using previous contributions [68] and the WHO framework, Figure 1 provides a theoretical model explaining the various pathways through which social deprivation can impact both maternal and newborn health. In our conceptual framework, we posit that annoyance caused by neighborhood conditions (and/or by socioeconomic position) may induce (or directly and/or indirectly enhance) some (psychological or physiological) disorders along the four hypothetical pathways described below. These four hypothetical pathways describe how social determinants can affect pregnancy outcomes and might lead to health inequality in utero and at birth: (i) Material circumstances; (ii) Maternal behaviors and lifestyle; (iii) Psychosocial environment; and (iv) Health system.

## 4. Pathway 1—The Mediating Role Played by Deprived Material Circumstances

Our first hypothesis is that both social neighborhood and the individual social position may affect pregnancy outcome as a result of unfavorable/deprived material circumstances. In fact, extensive literature reveals that in comparison with higher socioeconomic groups, those of lower socioeconomic levels live in less favorable material circumstances, including the physical environment in terms of, for example, environmental exposure (air pollution or noise, for instance), housing conditions and quality (of both the dwelling itself and its location), food availability and environmental amenities. Several studies have described social disparities in terms of the level of air pollution exposure, degree of access to green space, noise exposure, and availability of environmental amenities [68,69]. More specifically, deprived neighborhoods located in urban areas have fewer parks and walking trails as well as poorer access to green space in comparison with less deprived areas [70,71,72,73]. In addition to proximity to green spaces, their use depends on the level of education or income [74]. One explanation for this is that people living in deprived neighborhoods are less likely to use green spaces because they do not perceive the need to do so [75,76], though this has been challenged by other authors.

In addition, unfavorable circumstances could be interlinked (for instance, green space, air pollution, and noise [77,78,79]), leading to adverse health outcomes (including pregnancy outcomes). Many studies have revealed significant associations between various pregnancy outcomes (including LBW, PTB, and SGA) and deprived material circumstances such as air pollution (mainly particulate matter (PM)), nitrogen dioxide (NO_2_) [23,80], noise [81,82,83,84,85,86,87,88], green space [69,89,90,91,92], and environmental amenity [68].

According to our conceptual model, two pathways can explain how these deprived material circumstances (which are more prevalent in socially deprived environments) affect maternal and newborn health: a direct-action pathway via biological mechanisms, or physiological and psychological disorders, or an indirect-action pathway via unhealthy/healthy behaviors and lifestyle or psychosocial factors and a stressful lifestyle.

### 4.1. The Effect of Deprived Material Circumstances via the Direct-Action Pathway

The first direct-action pathway through which a deprived neighborhood can affect adverse pregnancy outcomes concerns biological mechanisms. For instance, the most plausible hypothesis relating to air pollution and adverse birth outcomes is that ambient air pollution could cause inflammation and oxidative stress, affect placental growth, reduce placental exchange, lead to endocrine disruption, etc. [93,94]. Oxidative stress can induce DNA damage (including mitochondrial DNA damage) as well as foster inflammation, and this appears to be an important fetal growth mechanism [95,96,97,98]. Another specific mechanism may affect the placenta; maternal and fetal circulation are separated by the placental barrier, which contains placental transporters capable of regulating (or facilitating) the circulation of external compounds [99,100]. Transient receptor potential channels are highly expressed in the placenta and can be affected by air pollution exposure. This hypothesis has been confirmed by non-human animal studies (mice model) showing that these receptors play important roles both in placental development and in regulating the fetal-maternal interface [101]. With regard to green spaces, there is evidence that a population’s perception of it directly impacts the human brain and body through psycho-neuroendocrine mechanisms, including hypothalamic–pituitary adrenal axis functioning, which regulates cortisol secretion and whose deregulation is associated with a range of adverse pregnancy outcomes. Other authors have shown that neighborhood characteristics (including recreational and resource levels) are associated with cardiometabolic pregnancy outcomes, such as the risk of impaired glucose tolerance during gestation and differences in postpartum weight retention [102].

*The second direct-action pathway posits that a deprived neighborhood can affect adverse pregnancy outcomes by operating through psychosocial disorders that cause stress.* The deprived neighborhood may have a positive impact (e.g., green space) or a negative one (e.g., noise exposure), diminishing or exacerbating psychological disorders that include stress, anxiety, and emotional and mental health. As proof of this, experimental studies have produced strong evidence of the positive effect of exposure to the natural world on recovery from stress and attention fatigue [69]. Other studies tell us that contact with natural environments promotes psychological restoration [103] and reduces stress and anxiety [104,105,106]. More generally, studies have shown lower neighborhood quality to be associated with a higher prevalence of depressive symptoms during pregnancy [107]. Poor neighborhood quality (defined by a high level of noise exposure, for instance) has been shown to play a role in the presence of stress [108]. Several studies found raised stress hormone levels (catecholamine, cortisol) in workers exposed to noise [109,110,111,112,113]. The effects of deprived material circumstances may thus reduce or increase maternal stress, with the support of neuroendocrine and immune mechanisms that may alter feto-maternal exchanges [108,114], causing limited fetal nutrition and/or oxygenation, and leading to both reduced fetal growth [115,116] and a higher risk of preterm birth [117].

*A third direct-action pathway through which a deprived neighborhood could alter a pregnancy is physiological disruption*—a term that refers mainly to sleep disorders such as insomnia, shorter sleep duration, and poor sleep quality. For instance, road traffic noise has been reported to be linked to a multitude of adverse health outcomes associated with physiological disruption, including annoyance [118], poor mental health [119], sleep disturbance [120], and cardiometabolic disorders [121]. More specifically, some studies report that sleep disturbance is significantly more widespread in urban populations exposed to traffic noise above 65 Leq dB (A) [122] and among populations living near airports [88]. Insomnia was found to be more prevalent among inhabitants living closest to busy highways [123], and some authors have suggested that some of these psychological disruptions (such as insomnia [124] and mental disorders [125]) are documented as being directly related to adverse pregnancy outcomes.

### 4.2. The Effect of Deprived Neighborhood via the Indirect-Action Pathway

In our conceptual framework, the indirect-action pathway posits that a deprived neighborhood could alter pregnancy outcomes through unhealthy behaviors and lifestyle, and/or physiological environment (the second and third parts of the “intermediary determinant component” are described below).

First, encouraging and offering opportunities for physical activity coupled with increased access to green spaces may improve maternal behaviors during pregnancy, with positive effects on physiological and metabolic disorders, including weight gain [126] and diabetes [127] during pregnancy. Some authors suggest that these physiological disruptions, such as cardiovascular conditions or obesity [128,129], are directly related to adverse pregnancy outcomes (this pathway is detailed further below). Many studies have linked access to green spaces with physical activity [76] and, in turn to adverse outcomes, via a number of underlying mechanisms, which are described below.

Second, offering improved access to healthy food (shown to affect diet quality during pregnancy) may improve the dietary habits of pregnant women, and this, in turn, may impact birth outcomes. For instance, women living more than four miles from a supermarket were twice as likely to fall into the lowest level of the diet quality index for pregnancy in comparison with women living within four miles of a supermarket [130,131].

Third, by fostering stressful life events and modifying social contact [132,133], the deprived neighborhood may impact pregnancy outcomes.

## 5. Pathway 2—The Mediating Role Played by Healthy Behaviors and Living Conditions

The second part of our theoretical model posits that poor social characteristics could be related to pregnant women’s unhealthy behaviors and lifestyles and that this could lead to a rise in the occurrence (or risk) of adverse health outcomes. Abundant literature shows that social deprivation (including education, income, and employment status) is associated with pregnant women’s behaviors and lifestyle, including diet, physical activity, smoking, and alcohol consumption [134,135,136]. Cohort studies of pregnant women have shown that moving from the lowest to the highest employment grades decreases tobacco consumption [134,135,137,138], and other studies have suggested that the most prominent health behaviors associated with educational disparities are smoking, followed by passive smoking [139,140]. Smokers who have benefited from higher education are more likely to stop smoking during pregnancy [141,142]. Most of these behaviors and lifestyles are more prevalent among socially deprived groups and are detrimental to health in relation to pregnant women; many studies have shown that maternal health lifestyle and health behaviors (such as smoking, alcohol consumption, and unhealthy nutrition) [135,143,144,145] have been associated with adverse pregnancy outcomes. Indeed, prenatal exposure to nicotine cigarettes (hereafter cigarettes), alcohol, cannabis, cocaine, and opioids, for instance, increases the risk of preterm birth, low birthweight, stillbirth, motor abnormalities, and mental health and cognitive problems [138,146,147,148,149,150,151,152,153,154,155].

Some studies reported on the relationship between pregnant women’s level of education and their behaviors during pregnancy, such as compliance with folic acid supplementation [156] and daily fruit consumption [157]. This is an important finding, given that we know that the use of multivitamins containing folic acid during pregnancy could significantly lower the risk of preeclampsia [158].

According to our conceptual model, two action pathways may explain how these unhealthy behaviors and lifestyles (more prevalent in socially deprived neighborhoods and/or positions) can affect maternal and newborn health: *(i)* biological mechanisms and *(ii)* physiological and psychological disorders.

Firstly, unhealthy behaviors and lifestyles could have an impact on adverse pregnancy outcomes through biological mechanisms. Goldstein et al. proposed a range of mechanisms to explain the effects of smoking during pregnancy; preterm birth may be due to vasoconstriction caused by smoking, which results in decreased blood supply to the fetus, reduced fetal nutritional supply, and a slower release of catabolism results. By increasing fetal CO levels and reducing both oxygen transport capacity and teratogenic properties, cigarettes may also have a direct toxic effect on the fetus [159,160].

Secondly, we posit that unhealthy behaviors and lifestyles may lead to physiological disorders (including obesity, gestational weight gain (GWG), and gestational diabetes) known to be related to adverse pregnancy outcomes. Several studies document the fact that women who are physically active during pregnancy are 24% less likely to develop gestational diabetes than inactive women [127], and in addition, the risk of spontaneous preterm birth falls [161]. Physically active pregnant women (whether the activity is light/moderate leisure-time or occupational) have a lower risk of developing preeclampsia [162,163,164], hypertension [162,164], and GDM [165,166,167]. More generally, meta-analyses have shown that regular physical activity improves both mental health and physical health in terms of blood pressure and chronic disease (breast cancer, colon cancer, diabetes, ischemic heart disease, and ischemic stroke events) [168,169,170].

Thirdly, through an association between physical activity [171,172], social contact [173,174], and mental health (including well-being, mood, and depression during pregnancy), unhealthy behaviors can affect psychosocial disorders, which in turn affect pregnancy outcomes by means of the mechanism described below.

Combined with unhealthy behaviors and lifestyles, both physiological and mental disorders can impact pregnancy outcomes. Many studies show maternal health conditions (such as obesity, low weight, stress, and depression [129,175,176,177,178,179]) to be associated with adverse pregnancy outcomes. For instance, high pre-pregnancy BMI and EGWG are associated with many unfavorable maternal and neonatal outcomes [180,181,182,183].

## 6. Pathway 3—The Mediating Role Played by Psychosocial Environment

The third part of our theoretical model posits that social deprivation may be related to many of the psychosocial environments (including life experience, isolation, the experience of racism, and social support) pregnant women find themselves in, and this could increase negative pregnancy outcomes. Many studies have documented the significant associations between psychological environment and various pregnancy outcomes, including PTB, LBW, and SGA. Both human and animal studies have confirmed that subjects exposed to stressors during pregnancy experience a greater risk of both spontaneous abortion and low birthweight [109,116,184,185]. For instance, 40% to 50% of women living in poverty are symptomatic for prenatal depression, and ethnic minority women living in poverty are twice as likely as white middle-class women to meet the diagnostic criteria for both major and minor depression [186,187]. For Hispanic and Black pregnant women experiencing depression, pregnancy and birth outcomes tend to be poorer than those of other pregnant women [188,189]. Pregnant Black women are also more affected by anxiety than women in the general population, and this can lead to poor maternal health and well-being outcomes, as well as adverse consequences for the health and development of their children [190,191,192,193].

Some studies suggest that the increased risk factors of adverse perinatal morbidity and mortality are proxies for social isolation, including teenage pregnancy [194], minority ethnic groups [195,196], the experience of domestic violence [197], asylum seekers, and refugees [198]. According to our conceptual model, two pathways could explain how these psychosocially deprived environments affect maternal and newborn health: through a direct-action pathway (via biological mechanism or psychological disorders) or through an indirect-action pathway (via unhealthy/healthy behaviors and lifestyle).

### 6.1. The Psychosocial Environment Effect: A Direct-Action Pathway

Adverse birth outcomes may also be related to the direct, biological adverse effects of anxiety, stress, or depression. Themselves are linked to psychological environment (including racism, social support, and isolation). One study showed that “unsafe space” was associated with a higher level of perceived stress and anxiety during pregnancy [68]. More specifically, Giurgescu et al. concluded that pregnant African American women’s negative perceptions of their own neighborhoods during the second trimester are associated with an increase in depressive symptoms during the last trimester [107,199]. Other studies have shown that preterm birth, low birth weight, and SGA risks were significantly higher for pregnant women living in unsafe spaces and reporting a high level of stress [68,176]. Biological mediators of increased anxiety, stress, and depressive symptoms trigger neuroinflammatory, neuroendocrine, and immune pathways. Through neuroendocrine and immune mechanisms, the pathway proposed in our model involves chronic stress (through activation of the central autonomic nervous system), triggering a series of biological events [200]. Maternal stress has been implicated in the production of catecholamine, cortisol, and inflammatory cytokines [109], which were found to be increased in both mother and fetus. The release of catecholamine may alter feto-maternal exchanges by increasing uterine contractions, blood pressure, and vasoconstriction of placental vessels and reducing uterine blood flow [108,114,201]. In turn, limited feto-maternal exchanges may affect fetal nutrition and/or oxygenation and, ultimately, fetal growth. Exposure to noise may therefore result in fetal asphyxia [115,116] and elicit both preterm birth and fetal growth restriction [202]. More recent research suggests that the Cortico-Releasing Hormone (CRH) stimulates the production of prostaglandin and oxytocin (which mediate uterine contraction); in this way, it can cause preterm labor [176,202,203].

Spontaneous preterm labor mechanisms include premature triggering of the fetalhypothalamic–pituitary axis, inflammation, matrix remodeling, abruption of the placenta, and mechanical stretch [204,205]. There is evidence that the perception of healthy spaces and safe spaces has a direct impact on the psycho-neuroendocrine mechanisms (such as hormonal and neuroendocrine changes) that may trigger a range of adverse pregnancy outcomes [185], including low birthweight, preterm birth, SGA, and smaller head circumference in infants [176,185].

Thus, by causing a psychological disorder (namely stress and anxiety), the psychosocial environment may play a crucial role in the occurrence of several adverse maternal and birth outcomes. Findings from a meta-analysis investigating the association between anxiety and birth outcomes have indicated an increase in the risk of preterm birth and low birthweight alongside a rise in anxiety [206]. In addition, in several studies, the prevalence of prenatal anxiety ranged from 16% to 54%, which has been related to poor health outcomes, including pre-eclampsia and excess weight gain [207,208,209,210]. The race could compound the effect of anxiety on birth outcomes since increased trait anxiety in Black women is related to preterm birth [191]. These findings are neither apparent in white women nor studied in Hispanic women. Some studies also suggest that postnatal depression could be associated with social isolation and inadequate support [211] and that women with antenatal depression (or a previous history of it) are at higher risk [212].

### 6.2. The Psychosocial Environment Effect: An Indirect-Action Pathway

Here, we posit that the psychosocial environment could, via an indirect-action pathway, alter pregnancy outcomes through unhealthy behaviors and lifestyle (a second aspect of the “intermediary determinant component” described above). Authors have suggested that people (and pregnant women in particular) living in unhealthy spaces are more likely to adopt unhealthy behaviors such as smoking, drinking alcohol, or eating a high-fat diet; these are known as coping mechanisms [69,123,199]. Such people may also be less likely to participate in healthy behaviors such as physical activity [73,76,89,213]. The psychosocial environment influences maternal health behaviors such as diet, sleep, and exercise. Unhealthy lifestyles and behaviors (including unhealthy eating or smoking) are often adopted as ways of coping with (or regulating) distressing emotions [213,214,215]. For example, unhealthy lifestyles (including unhealthy eating and a lack of physical activity) have been found to be more prevalent in Black people who are depressed and stressed [216]. In order to take these explanations of the mechanism involved one step further, many studies have suggested that anxiety, stress, and depressive symptoms are associated with poor lifestyle behaviors in pregnant women [187,217]. These unhealthy lifestyle behaviors may thus indirectly mediate the adverse effects of anxiety, stress, and depression on maternal and newborn outcomes. For instance, in pregnancy, EGWG [218], smoking [219], and poor nutrition [144] are all related to poor maternal health and birth outcomes [220,221].

## 7. Pathway 4—The Mediating Role of Access to Adequate Prenatal Healthcare Utilization

In terms of both timing and content, social deprivation could result in inadequate utilization of prenatal healthcare, and this, in turn, is related to adverse maternal and perinatal outcomes. Some studies found that inadequate antenatal healthcare is associated with severe adverse outcomes (especially for women from socially disadvantaged and ethnic minority groups) [222,223]. Another mechanism linking socioeconomic disadvantage to poor maternal and newborn health lies in its effect on healthcare access [224,225]. This difference in the utilization of healthcare (with health disparities outcomes for both mother and child) is observed even in countries offering free healthcare [223,226]. This leads to the hypothesis that disadvantaged populations (and, more specifically, the population of deprived pregnant women) may experience a greater diversity of barriers to healthcare utilization.

Based on the healthcare access framework developed in 2013 by Levesque et al., [227] a recent systematic review summarizing 37 studies focused on maternity care utilization and identified the barriers and facilitators affecting maternal healthcare utilization during the perinatal period among the population of socioeconomically deprived women [228]. With regard to the barriers and facilitators identified by Grand-Guillaume-Perrenoud et al. in the systematic review, we offer a few examples to describe how social deprivation can lead to inappropriate prenatal healthcare utilization, which in turn increases the risk of adverse pregnancy outcomes [228].

Firstly, social deprivation may affect prenatal care utilization due to a lack of information. The inability to find relevant and understandable information is a difficulty faced more frequently by socially disadvantaged women than by others [229]. Several studies have linked a lack of knowledge of available maternity services to care not being utilized [228,229,230,231,232].

Secondly, because of their low incomes, deprived women with financial difficulties are less able to pay for medications, immunization, or healthcare for their infants [233]. Some studies have suggested that unemployed women have been identified as a barrier to health care utilization [232], which is associated with late antenatal care (ANC) initiation [234,235] and inadequate ANC [236]. Some studies have also suggested that a lack of health insurance is associated with inadequate utilization of ANC [232,237].

Thirdly, social isolation [237], having few links within the community [232], and inadequate support networks [232,238] were identified as barriers to maternity care.

## 8. Public Health Intervention for Pregnant Women and Birth Health

**Figure 2:**
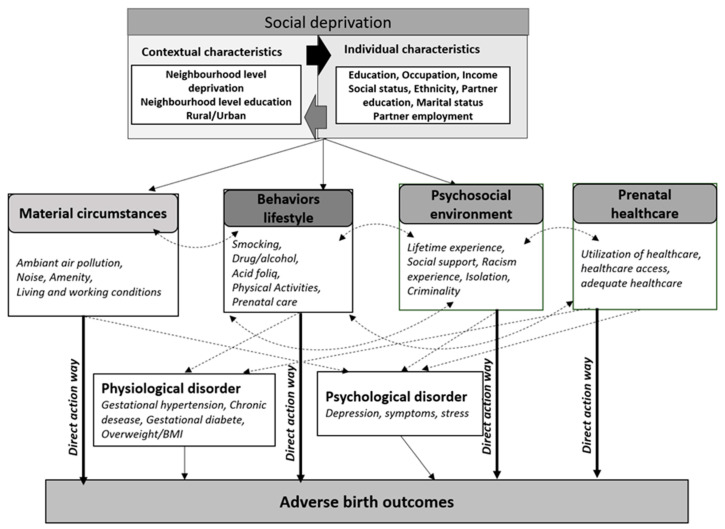
A conceptual model to explain the overlapping relationship between social deprivation (including individual and neighborhood level) and adverse birth outcomes.

Based on our conceptual model (described above), we make some suggestions for efficient interventions aimed at promoting maternal and newborn health. Relying mainly on randomized trial studies, we have distinguished three groups of interventions concerning: (i) Behaviors, lifestyle, and biological factors, (ii) Psychosocial factors and environment, and (iii) Public policies (see Figure 2).

(i). Behaviors, lifestyle, and biological factors

Antenatal health conditions and dietary and lifestyle interventions in pregnant women

As formalized in our conceptual model, both maternal health status (such as obesity) and individual behaviors (such as smoking and alcohol consumption or unhealthy nutrition) [135,143,144,145] are recognized as being related to adverse pregnancy outcomes, including PTB and LBW. In addition, these factors and behaviors are known to be more prevalent among poorer people. To promote maternal and newborn health, it, therefore, seems relevant to inform and better tailor and target interventions on the antenatal health and behaviors of pregnant women [239]. Previous studies (including meta-analyses and systematic reviews) have found that specific lifestyle interventions could improve some pregnancy outcomes in overweight and obese women [240].

Diet and physical activity during pregnancy

In 2015, following the Cochrane process [241], a literature review found that dietary intervention (combined or not with physical activity) during pregnancy is effective in preventing EGWG. Another systematic review evaluating dietary and lifestyle interventions in the population of pregnant women demonstrated a modest 1.25 kg difference in weight gain [242]. Its authors reported nine interventions consisting of face-to-face sessions with a trained professional [136,243,244,245,246,247,248,249,250]; their intensity ranged from three dietary sessions over the course of the pregnancy [248,249] to one at each antenatal visit [251]. In a comprehensive meta-analysis of 36 randomized trials involving more than 12,500 pregnant women, a modest effect on GWG was identified following dietary and physical activity advice (mean difference equal to 0.7 kg); there was very little effect on clinical pregnancy and neonatal outcomes [252]. One such randomized trial study by Bruno et al. [128] examined whether a one-hour individualized counseling session with a dietician resulting in the prescription of a hypocaloric, low-glycemic, low-fat diet, alongside physical activity and close monitoring, could have any effect on the incidence of GDM. These authors also sought to examine how compliance (in terms of the successful adoption of healthier eating habits) could influence this outcome and consequently be preventive in terms of both PTB and LGA. In the group that received both specific, personalized instructions and the diet and physical activity intervention, they found a decrease in the incidence of gestational diabetes in pregnant women with a BMI of ≥25 kg/m^2^, as well as decreases in the rate of PTB, induction, a number of babies too large for gestational age, and birthweight > 4000 g.

Unhealthy behaviors during pregnancy

Many public health interventions aim to reduce prenatal exposure to cigarettes, alcohol, cannabis, cocaine, and opioids. For instance, to improve child and maternal well-being, the US Nurse-Family Partnership (NFP) involves public health nurses providing frequent home visits from early pregnancy until children reach the age of 2 years [253,254].

In Canada, the British Columbia Healthy Connections Project RCT reports on how the NFP may impact the consumption of cigarettes, cannabis, street drugs, and alcohol use during pregnancy [255,256]. Nurses contacted pregnant women allocated to the intervention group to schedule the 14 prenatal visits, then assisted participants in identifying and meeting health and social goals, including (though not limited to) the reduction in prenatal substance use [257]. The authors found that, among pregnant smokers, the NFP significantly reduced both cigarette and cannabis use for participants. The NFP intervention may show promise in reducing certain types of prenatal substance use in disadvantaged populations and may also contribute to the reduction in negative birth outcomes.

(ii). Psychosocial and psychological interventions

Meta-analysis has shown that anxiety and other mental health problems during pregnancy can affect both newborn health and birth outcomes; specifically, the results indicated that an exacerbated state of anxiety was related to both preterm birth and low birth weight [206]. The implementation of preventive measures in antenatal health care aimed at reducing adverse pregnancy outcomes should, therefore, take both medical and non-medical risk factors into account. The Cochrane review [258] demonstrated that psychosocial and psychological interventions were more effective in preventing postnatal depression than run-of-the-mill care. As part of the ‘Healthy Pregnancy 4 All’ (HP4All) program in the Netherlands, a nationwide study assessed various strategies aimed at improving pregnancy outcomes, in particular among deprived populations [259]; this revealed that an intervention using the R4U scorecard to identify psychological, social, lifestyle, obstetric and non-obstetric care-related factors [260] improved the detection of potential preterm delivery and fetal growth restriction during pregnancy.

Perhaps it is intuitive to imagine that early and ongoing intervention on these stressors during pregnancy could improve health; several studies suggested the implementation of caseload midwifery care as an efficacy intervention aimed at promoting both maternal and newborn health. A crucial aspect of cascading midwifery care is the time required to identify needs and access social support services, in comparison with other, more traditional care [261]. The Cochrane review [262] showed that continuity of care (including caseload midwifery) reduced the risk of preterm birth (average risk ratio (aRR) = 0.7).

Many studies showed a significant reduction in preterm birth rates among pregnant women allocated to caseload midwifery in comparison to those receiving traditional midwifery care. Hadebe et al. confirmed this reduction in the PTB risk and also observed a reduced risk of birth by cesarean section in a deprived inner-city community, which could lead to a decrease in social health inequalities [263]. These interventions entail providing the time, continuity, and communication allowed by caseload midwifery care [264,265,266]. The time spent with the woman is intended to build trust [267], allow observation of where she lives, and assessment of risks that have not been fully verbalized so as to establish solutions to fit both her setting and her community [268,269].

(iii). Public policy

Behavior change communications (BCCs) have been used to improve maternal and child health practices, including in terms of nutrition [270,271,272]. Beyond these recommendations, however, broader access to information relating to pregnancy, preparation for childbirth, and best practice in caring for a newborn is crucial to promoting maternal and newborn health—especially among the most deprived populations. One option is the use of mobile health (mHealth), especially the use of texting (SMS), to accurately inform underserved women about best practices. One systematic review reported that almost 8500 health interventions conducted in low- and middle-income countries were being implemented via mobile phones (mHealth interventions) for maternal and child health [273]. An assessment of the effect of mHealth interventions on improving maternal and neonatal care and health showed that mHealth interventions targeting pregnant women would promote maternal and neonatal health service use [273,274,275]. More recently, Zhou et al. conducted a large-scale international study quantifying the impact of health texting via cell phone on birthweight in rural China. The authors revealed that a package of free informational text messages, including advice for good prenatal household practices and seeking care, could prevent inappropriate weight for gestational age.

## 9. Recommendations

Our conceptual model highlights the fact that—because the pregnancy period offers a great opportunity to address both the inequalities that begin in utero and the disparities observed at birth, as well as to preserve health capital when entering adulthood—it is important for an intervention targeting various unhealthy behaviors and conditions to be implemented in early pregnancy. Most interventions identified aimed to promote maternal and newborn health involving (either directly or indirectly) the antenatal model of care published by the WHO. Therefore, in order to foster healthy behavior and lifestyles (diet, sleep, smoking, and exercise) and/or ensure a healthy psychological and social environment (in terms of social support, isolation, and anxiety), it seems both relevant and effective to include both healthcare setting and caseload midwifery. Health policy should support midwifery availability as well as accessibility and enhanced multidisciplinary support for deprived pregnant women. The targeted continuity of midwifery care dedicated to poorer pregnant women and/or those living in deprived areas may be suggested as a fitting intervention with which to address the social inequalities related to pregnancy outcomes.

Caseload midwifery refers to the continuity and booking of midwifery care from the antenatal period through to the postnatal period, with appointments (including home visits) gradually becoming longer and more frequent during this vulnerable time. Both the time spent and the communication with women are particularly important to understanding their risk perception, their knowledge about risks (to their own health and that of their child), and their general level of confidence in the health system. They also allow observation of their living conditions to assess real risks and thence identify options capable of minimizing the health risk [263].

The intervention at the crux of caseload midwifery care is the provision of time, continuity, and communication [264,265,266]. Time spent with a woman serves to build trust and rapport [267] and provides an opportunity to observe her surroundings, assess risks not verbalized, and come up with solutions tailored to fit the woman’s setting and community [268,269].

Despite the known cost-effectiveness of caseload midwifery intervention in several countries, it has yet to be systematically implemented for all women. A scoping review of randomized trials and observational studies comparing antenatal midwifery to physician-led care for women of low socioeconomic status has confirmed a reduced risk of PTB, LBW, and/or VLBW for midwifery patients [269].

The involvement of different institutions leads to multidisciplinary consultations, and this, in turn, favors a more proactive and preventive approach to all intermediate determinants in the course of pregnancy—including behavior, lifestyle, and psychosocial factors. Designing an intervention for the poorest pregnant women in which multidisciplinary consultations take pride of place demands the involvement of community midwives, obstetricians, and other health professionals such as pediatricians, diabetics, or social workers. For this approach, optimal links will be pursued between the public health sector and the curative care sector. The aim of this intervention is to agree on a customized antepartum policy for each pregnant woman, with particular attention to those most deprived. This preventive strategy is currently poorly implemented—yet in the Netherlands, as part of the HP4All program, the authors of the experimental study have suggested that the implementation of additional multidisciplinary consultations in general practice is both feasible and effective and needs to be more widely implemented. The main results of this review are summarized in Table 2. 

## 10. Conclusions

Using a comprehensive review, we summarize the effect of socioeconomic characteristics (at individual and neighborhood levels) on pregnancy outcomes, as revealed in the selected studies addressing social inequalities in adverse birth outcomes. On the basis of 35 papers, our review reveals an excess risk of both LBW and PTB among deprived pregnant women and/or newborns living in deprived neighborhoods.

In the second part of our paper, to understand the mechanisms of these social inequalities, we present a conceptual framework based on the work of both the Commission on Social Determinants of Health and the WHO. Using our adapted WHO framework, we describe the different pathways through which social characteristics may affect both pregnancy and fetal health, with a focus on how structural social determinants (such as the socioeconomic and political contexts that influence social position) affect intermediary determinants. Using a more detailed framework, we then propose a theoretical contribution aimed at gaining a better understanding of the potential effect of social deprivation on maternal and newborn health. Our model suggests that, based on their social position, individuals may experience differences in environmental exposure and vulnerability to health-compromising living conditions. To address both the inequalities that begin in utero and the disparities observed at birth, it is thus important for interventions to target various unhealthy behaviors and psychosocial conditions in early pregnancy. In conclusion, health policy needs to support: *(i)* midwifery availability and accessibility and *(ii)* enhanced multidisciplinary support for deprived pregnant women.

## Figures and Tables

**Table 1:** Main characteristics of the selected studies.

Author	Study Design Period Localisation	Population	Outcome	Deprivation Scale	Level Zone	Confounders	Modèle/Analyse Stat
Enstad et al., 2019 [26]	Cross-sectional study, Infant from Illinois (1989–1991) from parents born in Chicago (1956–1976)	singleton births of African American (n = 8331), non-Latina White (n = 18,200), and Latina (n = 2637) women	SGA	Maternal SES - Maternal education, Paternal SES -contectuel median family income of father’s census tract residence during childhood and parenthood	Individual level and Census tract	Ethnicity Maternal age Marital status	Stratified and multilevel, multivariable logistic regression analyses
Ospina et al., 2020 [28]	Cross-sectional study, Alberta, Canada (2006–2012)	Women (n = 330,957) with singleton births	PTB, SGA; LGA,	2006 SES index area-based socioeconomic gradients	Census block	Rural(CIdxR)/Urban(CIdxU), smoking and substance use during pregnancy and prepregnancy weight >91 kg gestational hypertension, gestational diabetes, maternal age at delivery	Prevalences comparison accross SES quintiles by calculating a absolute concentration index
Wilding et al., 2019 [58]	birth cohort Southampton, UK (2004–2016)	singleton births (n = 65,909)	SGA	Maternal SES - education - employment - partnership	Individuel level	Maternal smoking, BMI, parity. maternal age, parity, ethnicity, gestational diabetes, gestational hypertension and systolic blood pressure at booking	Multivariable logistic regression
Melissa et al., 2016 [57]	4 nationally representative Cohorts study United states, United Kingdom, Canada and Australia (1998–2004)	singleton birth United States (n = 8400), the United Kingdom (n = 12,018), Canada (n = 5350), and Australia (n = 3452)	LBW	Maternal SES - maternal education Neighborhood SES Income quintile Income quintile calculated from total family income, available in each country, adjusted for family size	Individual level/country level	Maternal age Marital status Parity, Mother’s nativity, child’s sex, prenatal smoking race/ethnicity and region of origin	logistic regression models
van den Berg et al., 2012 [50]	Cohort study in the Netherlands, the Amsterdam (2003–2004)	Pregnant women (n = 3821)	PTB LBW SGA	Maternal SES maternal educational attainment The number of years of education after primary school was obtained by questionnaire, and categorized as low (less than 6 years of education after primary school), mid (6 to 10 years) and high (more than 10 years).	Individuel level	maternal smoking sex, maternal age, maternal height, parity maternal pre-pregnancy body mass index	logistic regression analysis
Patil et al., 2019 [29]	Cohort study USA (1979–2014)	Pregnant women (n = 2871)	LBW	Maternal SES Employment precarity scores evaluated using availability of employer sponsored insurance, income, long shifts, non-daytime shifts, availability of employer-sponsored training or educational benefits and membership in a union or collective bargaining unit	Individual level	maternal age, race/ethnicity, educational attainment, nativity, prepregnancy body mass index, alcohol consumption, smoking during pregnancy and infant year of birth	Modified Poisson regression models
Elo et al., 2009 [30]	The birth records based study Baltimore City, Baltimore County, Montgomery County and Prince Georges County in Maryland, 16 pooled cities in Michigan, Durham County and Wake County in North Carolina, and Philadelphia, Pennsylvania USA (1995–2001)	NA	SGA	neighborhood-level deprivation index (income/poverty, employment, education, housing, and occupation.) race/ethnicity	residential census tracts	Maternal age, maternal education, mother smoked during pregnancy, gestational and/ or chronic hypertension	Multilevel random intercept logistic regression models
Misra et al., 2010 [31]	a hybrid retrospective and prospective cohort Baltimore, Maryland USA (2001–2004)	women with singletons birth (n = 832)	PTB	Maternal SES Education level Family resource scale	Individual level	Stress depression symptoms pregnancy locus of control mastery anxiety and social support maternal age, education, income, and the Family Resources Scale cigarette smoking, alcohol and illicit drug use, and vaginal douching, parity, multiple gestation, initiation of prenatal care, number of prenatal visits, chronic diseases and complications of pregnancy	Cox proportional hazards analysis
Pei et al., 2015 [56]	cross-sectional study Shaanxi province China (2010–2013)	singleton births (n = 28,722)	LBW Macrosomia	Maternal SES education (primary, secondary and _ high education),employment (farming and other occupations which included teacher, official, commercial and service staff, and professional), Demographic and Health Survey household wealth index (HWI) (5 variables of family economic level: housing conditions, type of vehicle, income resources, and type and number of household appliances)	Individual level Household	sex, prematurity gestation (weeks) maternal age maternal health conditions negative (adverse) life events alcohol intake and passive (secondhand) exposure to smoke month antenatal care the number of ANC visits folic acid supplementation	generalized linear model
Sims et al., 2007 [32]	Vital Records Birth based study Wisconsin USA (1998–1999)	Pregnant women (n = 100,074). African-Americans (n = 11,313) Latinos (n = 6450)	LBW VLBW	Maternal SES Education level Neighborhood SES community-level income	Individual and community-level by zip code	individual-level characteristics individual-level factors	Multinomial logistic regression analysis
Glinianaia et al., 2013 [51]	hospital neonatal records based study Newcastle upon Tyne, North of England, (1961–2000)	singleton births (n = 113,182)	Birthweight LBW PTB	neighborhood SES Townsend Deprivation Score (proportion of home ownership, car ownership, unemployment and overcrowding)	Enumeration district (ED)	Gestational age Maternal age, parity and infant sex decades of birth	linear regression logistic regression
Kaufman et al., 2008 [33]	cross-sectional study Santiago Chile (2004)	Singleton births (n = 56,970)	PTB	Maternal SES Maternal Years of Education Neighborhood SES inverse density (i.e., number of domiciles per capita in the district) percentage of homes connected to a sewer system logarithm of the average total valuation per square meter percentage of the population that does not self-identify as indigenous percent of the population with formal schooling percent of the population that is not currently unemployed and seeking work percentage of the population that is classified as an owner or employer percent of domiciles that have concrete paving percent of domiciles that have indoor plumbing the percent of domiciles that have indoor heating.	Census district/individual level	parity, sex of child and maternal age	multilevel regression analyses logistic regression
DeFranco et al., 2008 [34]	Cross sectional study Missouri USA (1989–1997)	Singleton births (n = 634,994)	PTB	Individual-level SES maternal and paternal highest educational attainment and marital status (Mother’s education level < high school) The area-level SES County-level poverty rates included the percentage of the population within each county living below the poverty line	Individual/Counties level	Demographic factors Maternal age Reside inside city limits Black race Married Prenatal Care Inadequate prenatal care Behaviors Maternal tobacco use Maternal alcohol use Medical Risk Factors Medical risk factors	multilevel logistic regression analysis
Young et al., 2010 [35]	retrospective cohort study Cape Cod, Massachusetts (1969–1983)	singleton births (n = 1689)	Birthweight	Maternal SES maternal education; paternal occupation; Comunity level percent adults living in poverty; percent adults with a four years college degree; community mean family income; and percent adult unemployment	Individual, family- and community-levels (Enumeration district level)	child gender, gestational duration, birth order, year of birth, maternal age and race, adequate prenatal care, inadequate maternal weight gain, and cervical incompetence during pregnancy, any history of maternal diabetes or hypertension, prior low birth weight or preterm infants, and maternal smoking during pregnancy	multilevel models
Urquia et al., 2011 [36]	population-based study Ontario Canada (2002–2007)	singleton births (n = 474,614)	PTB	neighborhood deprivation index material-deprivation score: percent of population below the Statistics Canada low income cutoff, percent of population 20 years and over without high school diploma, percent of single-parent families, percent of income comprised of government transfer payments, percent of population unemployed and percent of homes needing major repairs) immigration Maternal SES Mother educational level	census tract level	Sex maternal age parity language knowledge maternal country of birth, age at arrival graduation marital status immigrant class knowledge of either official Canadian language	cross-classified random effect models
Kozhimannil et al., 2013 [37]	Cohort study USA (2005)	women with singleton birth (n = 1573)	LBW PTB	Maternal SES prenatal employment status (full-time, part-time, not employed)	Individual level	age, education, race, region, marital status, unintended pregnancy, mistimed pregnancy, fertility treatment, priorcesarean delivery, interaction between race and parity, interaction between parity and region, and interaction between age and marital status.	multivariable regression models.
Filho et al., 2007 [38]	Cohort study, Ribeirão Preto (1978–1979 and 1994) São Luís (1997–1998) Bresil	Singletons birth in Ribeirão Preto (n = 6747) in São Luís (n = 2839)	LBW PTB SGA	Individual SES family income, maternal schooling, occupation of the head of the family, paternal schooling, and marital status of the mother.	Individual level	gestational age were birth weight, parity, family income, and newborn infant sex	regression model
Wallace et al., 2013 [39]	Birth records based study Bogalusa USA (1987–2000)	women-with singleton births (n = 2743)	LBW PTB	Neighborhood SES poverty (percentage of households living below the federal poverty level + then categorized by their race and poverty level of the neighborhood) Individual level Maternal SES education at time of first birth (less than high school, high school, greater than high school)	Individuel/U.S.Census block group	Stratified by race// maternal, age, smoking during pregnancy, year of BHS examination and years between examination and conception	Generalized estimating equations
Mason et al., 2010 [40]	Birth records based study New york city USA (1995–2003)	singleton births (n = 925,277)	PTB	Maternal SES Education level education taking age into account (indicators for <12 years and age <20 years, <12 years and age _20 years, 12 years, 13–15 years, and _16 years), parity (indicators for 1, 2–5, and _6 previous births),Neighborhood SES residential stability and neighborhood deprivation the percentage of the neighborhood population residing in the same house from 1995 to 2000. Neighborhood deprivation was represented by using a standardized index arising from17 tract-level census variablesneighborhood immigrant or ethnic density in area of residence	Individual and census tracts (median area of 0.18 km^2^)	maternal age education taking age into account parity tobacco use prepregnancy weight payment type	logistic regression
Colen et al., 2006 [41]	Cross sectional study USA (1979–2002)	White Women with singletons birth (n = 574 = and Black Women with singletons birth (n = 1270)	LBW	Second-generation maternal SEP during adulthood. Maternal SEP Family income - Chronically poor was defined as living in a household during both childhood and adulthood where the income-to-needsRatio ≤ 200% of poverty. - Upwardly mobile is defined as living in a household during childhood, but not adulthood, where the income-to-needsratio≤200% of poverty Second-generation maternal SEP during childhood. The NLSY79 contains information concerning the occupation and educational attainment of first-generation individuals but does not provide measures of income	Households level	inflation using the Consumer Price Index for All Urban Consumers, Experimental Series, and reported in 2002 dollars race and gender	multivariate analyses logistic regression models.
Mortensen e at al., 2008 [52]	Cross sectional study Denmark, Finland, Norway and Sweden (1981–2000)	singleton birth (n = 1,077,584) Finland n = 400 442; Norway n = 929,458; Sweden n = 1,761,562).	Birthweight SGA LGA	Maternal SES - Mother education (years) - father’s education (years)	Individual level	r gestational age, parity, mother’s age, whether a father was known, father’s education and father’s age.	Linear and binomial linear regression
Dunlop et al., 2021 [42]	Cohort study USA (2018)	Women with singleton birth (n = 25,526)	PTB	Maternal SES Maternal level of education Neighborhood-level SES Urbanicity percentage black population d percentage population living below the 100% federal poverty line Maternal race and ethnicity	Individuel and Census tract	maternal age maternal parity marital status child sex maternal prenatal body mass index type 1 or type 2 diabetes mellitus; chronic hypertension; chronic infections (including HIV and hepatitis B or C); other chronic health conditions including asthma, other lung disease, cardiac disease other than hypertension prenatal tobacco use, alcohol use, and use of marijuana, stimulants, opiates pregnancy complications, gonorrhea, trichomoniasis, cervicitis, and pelvic inflammatory disease; receipt of prenatal care (yes/no) and prenatal health insurance	Multinomial logistic regression
Majdan et al., 2018 [53]	cross-sectional study Slovakia (2009–2013)	Municipalities (n = 2515)	LBW	Neighbodhood SES municipalities with minor Roma population municipalities with large Roma population Rate of unemployment	Municipalities level	Mean age of mothers at the date of birth and proportion of registered unemployed people in the respective municipality	square regression models multivariable models
Vang et al., 2013 [43]	Cross sectional study New Jersey USA (2002–2006)	Singleton births (n = 73,907)	Birthweight	- neighborhood minority diversity standardized entropy score - Neighborhood Deprivation - Residential instability Maternel SES educational level	Individuel and census tract	maternal health behaviors and conditions, and gestational age	linear regression
Huang et al. 2015 [45]	Cohort study USA (1995–2009)	Women with singleton birth (n = 1681)	Birthweight	Grandmaternal educational level Maternal education level	Individuel level	maternal life-course factors: maltreatment as a child, education and income as an adult, prepregnancy overweight, and prenatal smoking	marginal structural model (MSM) approaches
Shankardass et al., 2014 [46]	Cross sectional study Nova Scotia Canada (1988 and 2003)	singleton births (n = 117,734)	SGA LGA PTB perinatal death post-neonatal death	Maternal SESTotal family income Neighborhood deprivation index of neighborhood deprivation (None information)	Family (individuel) and Postal code	urban or rural place of residence and birth year marital status parity, pre-pregnancy weight, weight gain during pregnancy, maternal age, maternal smoking at delivery gestational diabetes and prenatal class attendance.	Multiple logistic regression
El-Sayed et al. 2012 [47]	Cross sectional study Michigan USA (1989 2006).	Pregnant women (n = 1,876,471)	PTB	Maternal SES maternal education	Individuel level	race maternal age at parturition ( parity stratified by year and analyzed births by year of occurrence.	bivariate and multivariable Poisson regression models
Ryu et al., 2016 [59]	cross-sectional study Olmsted County, Minnesota Jackson County, Missouri USA (2006)	parents (n = 728) with singleton birth (n = 701)	LBW	SES measure based on 4 housing-related characteristics (termed HOUSES) housing-related characteristics HOUSES index - parental education level - family annual income - Hollingshead index - Nakao-Treas index square footage of housing unit, (2) assessed housing value, (3) number of bathrooms, (4) number of bedrooms, (5) ownership of housing unit, (6) residential status and (7) lot size of housing unit in acres, and six neighborhood characteristics collected from census tract-level data, including (1) per cent of people speaking English as a second language, (2) per cent of foreignborn people, (3) per cent of households headed by a female, (4) per cent of households that are non-family households, (5) per cent of people with less than a high school education and (6) per cent of families with family income below poverty level.	Census tract level ? house level ??	smoking exposure	gradient boosting machine (GBM) models underlogistic regression model framework
Raab et al. 2022 [54]	Cohort study Bavaria Germany (2013–2015)	Pregnant women (1738)	PTB	Educational level	Individuel level	maternal age, parity, and pre-pregnancy BMI.	Multivariate logistic regression models
Pardo-Crespo et al., 2013 [48]	Cross sectional study Olmsted County, Minnesota USA (2000)	Households (n = 750)	LBW Overweight	individual-level SES - Parents’ highest education level - annual family income Area-level SES - percentage of people with a bachelor degree or higher education - median family income at a census block-group level.	Individual and Census block group level	tobacco smoking status of household members	Cohen’s κ indexes using the categories of individual-level and arealevel SES measures logistic regression models
d’Orsi et al., 2005 [49]	Ecological study Rio de Janeiro Brasil (1991–1994)	singleton births (n = 97,519)	LBW	socioeconomic index Mean percentage of: - single-family buildings - sewerage access b - rented domiciles - income >10 x min. wage - slum or “favela” census tracts	Census tract level (as neighborhood)		Spatial analysis, multivariate cluster classification non-hierarchical clustering K-means algorithm Moran “I” statistics
Dičkutė et al., 2004 [55]	Case-control study Lithuania (2001–2002)	851 singleton births with LBW (n = 851) Singleton births control (n = 851)	Birthweight LBW	Maternal SES - Education level (primary.secondary, university) - Income - employment status (before and during pregnancy)		Smoking, alcohol consumption and drug use during pregnancy	univariate analysis logistic multivariable regression analysis

PTB was defined as a delivery from 24 through 36 weeks of gestation. LBW was defined as a weight below 2500 g. Newborns were categorized as SGA if they had a birth weight below the 10th percentile for gestational age on the basis of sex- and parity- specific standards.

**Table 2:** Main results of this work.

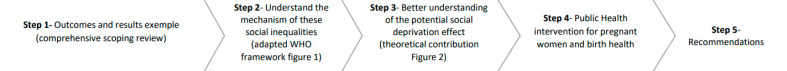						
**LBW**	Low maternal level of education	OR = 2.0 [1.49;2.71] [55]	**Structural determinant**: **Context** (governance, social policies, public policies) **Social position matters** (income, education, occupation) **Intermediary determinant:** Heath system Material circumstances Psychosocial factors Behavioral/biological factors	**Four Pathways proposed with direct and indirect action:** **Pathway 1**- The mediating role played by deprived material circumstances. **Pathway 2**- The mediating role played by healthy behaviors and living conditions **Pathway 3**- The mediating role played by psychosocial environment **Pathway 4**- The mediating role of access to adequate prenatal healthcare utilization	**Example of some efficient interventions:** **Antenatal health conditions and dietary and lifestyle interventions:** o Diet and physical activity during pregnancy o Unhealthy behaviors during pregnancy **Psychosocial and psychological interventions** **Public policy**	**Suggestions of a targeting intervention**: Interventions to target various unhealthy behaviors and psychosocial conditions in early pregnancy. health policy must support: *(i)* Midwifery availability and accessibility *(ii)* Enhanced multidisciplinary supports for deprived pregnant women.
		RR = 3.77 [2.26;6.30] [38]				
	Unemployment during pregnancy	OR = 1.7 [1.37;2.10] [55]				
	Low maternal income	OR = 1.7 [1.58;2.84] [55]				
	Bottom income quartile	In US: OR = 2.37 [1.80;3.11] [57]				
		In UK: OR = 1.78 [1.30;2.44] [57]				
		In Australia OR = 2.11 [1.12;3.99] [57]				
	Low level of employment precarity	RR = 1.48 [1.11;1.98] [29]				
	African-American women living in low-poverty	OR = 5.23 [2.26;12.10] [39]				
**PTB**	Highest quartile of poverty compared to the lowest quartile	OR = 1.18 [1.03,1.35] [34]				
	Deprived neighborhoods	OR = 1.16 [1.06;1.27] [46]				
	Most deprived groups	OR = 1.5 [1.3;1.7] [51]				
**SGA**	Most deprived neighborhoods	OR = 1.18 [1.07;1.30] [46]				
	Lowest income decile	OR = 2.0 [1.78;2.26] [46]				
	Head of family had a less skilled occupation	RR = 1.79 [1.29;2.47] [38]				

OR: Odd ratio, RR: relative risk.

## Data Availability

Not applicable.
